# Immunomodulatory and Anticancer Activities of *Hyacinthus orientalis* L.: An In Vitro and In Vivo Study

**DOI:** 10.3390/plants10040617

**Published:** 2021-03-24

**Authors:** Lina T. Al Kury, Zainab Taha, Wamidh H. Talib

**Affiliations:** 1 Department of Health Sciences, College of Natural and Health Sciences, Zayed University, Abu Dhabi 144534, United Arab Emirates; zainab.taha@zu.ac.ae; 2 Department of Clinical Pharmacy and Therapeutics, Applied Science Private University, Amman 11931, Jordan

**Keywords:** plant extracts, anticancer, immunomodulatory, traditional medicine, apoptosis

## Abstract

*Hyacinthus orientalis* L. (family Hyacinthaceae) is traditionally used to treat different diseases including cancer. In this study, the anticancer and immunomodulatory effects of this plant were evaluated. Hydroalcoholic extract was prepared, and different solvent fractions were obtained using solvent–solvent extraction. In the anticancer part, MTT assay and caspase-3 ELISA kits were used to measure the antiproliferative and apoptosis induction ability for each extract, respectively. In the immunomodulatory part, lymphocyte proliferation assay and cytokines detection kit were used to measure the effect of extracts of acquired immunity. Phagocytosis and pinocytosis induction were used to evaluate the effect of extracts on the innate immunity. GC–MS, LC–MS, and Foline–Ciocalteu assays were used to identify the chemical composition of the plant. Balb/C mice were inoculated with breast cancer and treated with hydroalcoholic extract of *H. orientalis* L. Results showed that hydroalcoholic extract and *n*-hexane fraction were highly effective in apoptosis induction. Both extract and fraction were also effective in stimulating lymphocytes proliferation and phagocytosis. Significant reduction in tumor size was achieved after treating tumor-bearing mice with hydroalcoholic extract. Additionally, high cure percentages (50%) were obtained in treated mice. Results of this study showed that *H. orientalis* L. has promising anticancer and immunomodulatory activities. However, further studies are needed to explore more details of apoptosis induction ability and other mechanisms of action and to measure different signaling pathways responsible for the anticancer and immunomodulatory response.

## 1. Introduction

Cancer is a leading cause of death worldwide. According to recent World Health Organization (WHO) reports, cancer is ranked as the first or second leading cause of death in 172 countries. Estimates for 2018 showed a global cancer incidence of 18.1 million cases with 9.6 million deaths [1]. Conventional cancer therapies (surgery, radiation, and chemotherapy) have their disadvantages. The use of chemotherapy is associated with cancer recurrence, emergence of resistance, and toxicity to the immune system and normal cells [2]. Therefore, finding new medications with low toxicity and higher activity is a priority in cancer research.

Plants represent an unlimited source of biologically active natural products that exhibit high potential to target cancer with relatively limited toxicity. However, the use of many plant-derived anticancer agents is associated with toxicity and development of drug resistance. Estimates showed that around 70,000 plant species are used for medical purposes [3]. Natural products are also gaining significant attention as effective anticancer agents. Exploring the ability of plants to treat cancer revealed more than 3000 species with anticancer properties [4]. Currently, more than 60% of anticancer agents originate from plants [3]. Examples include vincristine and vinblastine (from *Catharanthus roseus*), Taxol (from *Taxus brevifolia*), Camptothecin (from *Camptotheca acuminate*), and Harringtonine and Homoharringtonine (from *Cephalotaxus harringtonia*) [5]. The anticancer effect of these natural products is mediated by different mechanisms, including apoptosis induction, immune system modulation, and angiogenesis inhibition [6].

The use of herbal medicine by cancer patients is a common practice. In the USA, more than 35% of cancer patients are using herbal medicine with chemotherapy [7]. Additionally, herbal medication is effectively used by cancer patients in the UK for multiple purposes, such as preventing or relieving some of the direct symptoms of the cancer itself or dampening down the direct side effects of chemotherapy and radiotherapy used in the initial treatment process [8]. Despite this, there have only been a limited number of studies to evaluate the biological activity of a broad spectrum of herbs traditionally used to treat cancer.

*Hyacinthus orientalis* L. is an ornamental plant belonging to Hyacinth genus of the Asparagaceae (formerly, Hyacinthaceae) family. Hyacinth is a small genus of bulbous herbs native to the Mediterranean region and tropical Africa. Additionally, it is widely grown in Turkey, France, and the Netherlands [9,10]. In traditional medicine, the whole plant, leaves, and bulbs of *H. orientalis* L. were used as hemostatic, for the treatment of prostate disease and hemorrhoids and wound healing [9]. Although limited studies have tested the biological activities of this plant [11,12,13,14], chemical analysis revealed the presence of nine acylated anthocyanins in the flowers of *H. orientalis* L [15]. Anthocyanins are well-known for their antioxidant, anti-inflammatory, and anti-mutagenesis effects [16]. In addition, compounds isolated from this plant showed potent inhibitory activity toward α-glucosidase, and therefore have the potential to be used in the treatment of diabetes [17].

## 2. Results

### 2.1. Moisture Content and Percentage Yields of H. orientalis Hydroalcoholic Extract and Fractions

Determination of moisture in powdered plant material revealed that the moisture content in this plant is 9%. The percentage yield resulted from maceration was 24.3% of hydroalcoholic extract. On the other hand, fractionation method produced the following percentages: the highest yield was for aqueous fraction (46%), followed by chloroform fraction (20.7%), and n-hexane fraction (12%). (Table 1)

### 2.2. Antiproliferative Activity of H. orientalis L. Extracts

**Figure 1:**
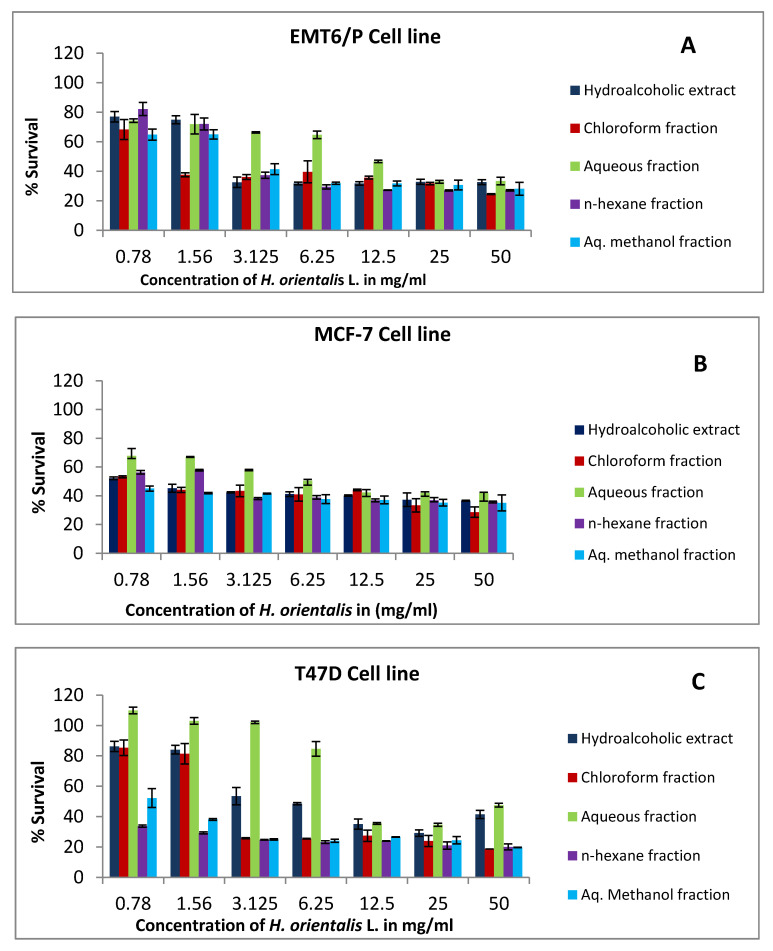

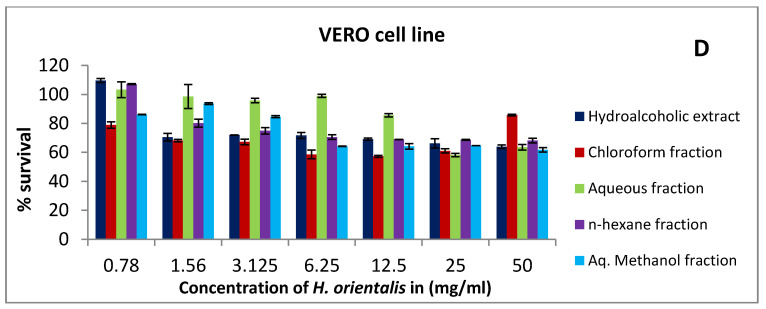
The antiproliferative activity of *H. orientalis* L. extract and fractions against: (**A**) EMT6/P cell line, (**B**) MCF-7 cell line, (**C**) T47D cell line, (**D**) Vero cell line using concentrations between 0.78 to 50 mg/mL. Percentage of cell viability (%) was calculated as (OD of treated cells/OD of control cells * 100). Results are expressed as means of three independent experiments (bars) ± SEM (lines).

The effects of various concentrations of *H. orientalis* L. extracts on the proliferation of EMT/6, T47D, and MCF-7 cell lines are shown in Figure 1. Hydroalcoholic extract and chloroform fraction were more cytotoxic against MCF-7 cells with IC₅₀ values of 0.95 and 0.70 mg/mL, respectively. However, aqueous methanol and n-hexane fractions exhibited high activity against T47D cells with IC₅₀ values less than 0.7 mg/mL, respectively (Table 2). Variable degrees of inhibition were observed in MCF-7, T47D, EMT6, and VERO cell lines with lower toxicity toward VERO cells (Figure 1, Table 2).

### 2.3. Apoptotic Activity of H. orientalis L. Extract and Fractions against T47D Cell Line

**Figure 2:**
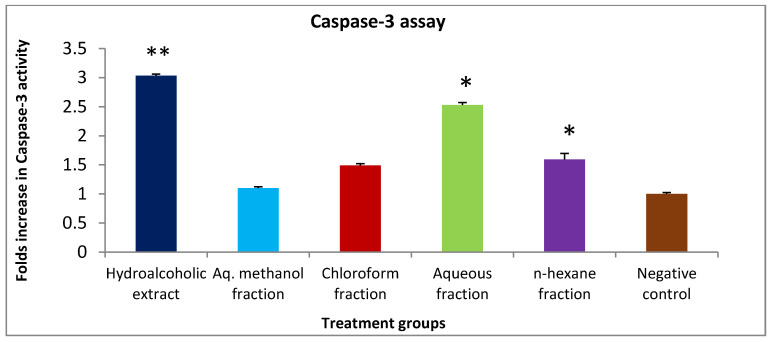
The effect of IC₅₀ concentration of *H. orientalis* L. extract and fractions on caspase-3 expression in T47D cell line. Concentration of the extracts: hydroalcoholic (6.12 mg/mL), aqueous methanol (0.11 mg/mL), chloroform (3.53 mg/mL), aqueous (11.38 mg/mL), and *n*-hexane (0.59 mg/mL) (* *p* < 0.05, ** *p* < 0.001 compared to the negative control). Results are expressed as means of three independent experiments (bars) ± SEM (lines).

Caspase-3 activity assay was used to measure the ability of *H. orientalis* L. to induce the programmed cell death. Hydroalcoholic (6.12 mg/mL), chloroform (3.53 mg/mL), aqueous (11.38 mg/mL), and *n*-hexane (0.59 mg/mL) significantly enhanced caspase-3 activity. Both hydroalcoholic extract and aqueous fraction enhanced caspase-3 activity by 3- and 2.5-fold of the negative control, respectively (Figure 2). However, aq. methanol fraction had no effect on caspase-3 activity.

### 2.4. Effect of H. orientalis L. Extract and Fraction on Lymphocytes Proliferation in the Presence and Absence of Mitogens

**Figure 3:**
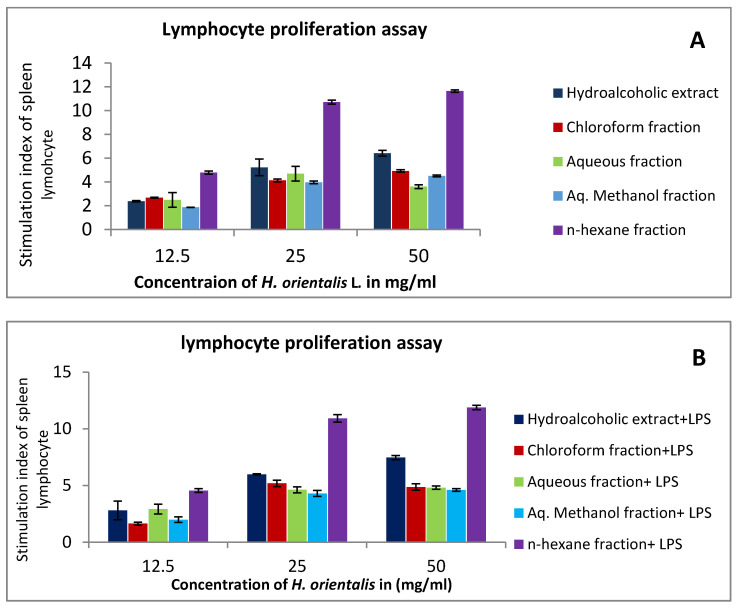

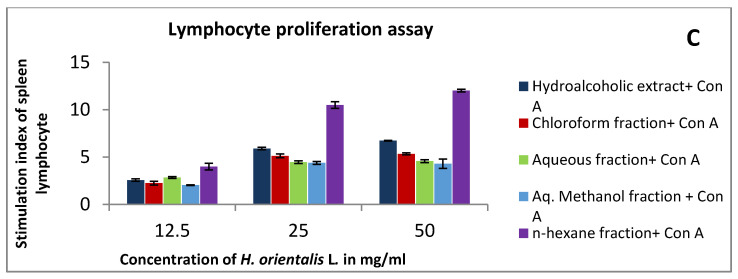
The effect of *H. orientalis* L. extract and fractions on the splenic lymphocytes’ proliferation using different concentrations (12.5–50 mg/mL) in the presence and absence of mitogens: (**A**) in the absence of Con A (concanavalin A) and LPS (lipopolysaccharide); (**B**) in the presence of (4 μg/mL) of LPS; (**C**) in the presence of (5 μg/mL) of Con A. Results are expressed as means (bars) ± SEM (lines).

At a concentration of 50 mg/mL, *n*-hexane and hydroalcoholic were the most active extracts in the presence and absence of mitogens. Stimulation index of *n*-hexane fraction and hydroalcoholic extract were around 12 and 7.4, respectively, in Con A and LPS-stimulated cells. On the other hand, in the absence of mitogens, the stimulation index of *n*-hexane fraction and hydroalcoholic extract were 11.6 and 6.4, respectively (Figure 3). Other solvent fractions showed variable activities.

### 2.5. Effect of H. orientalis L. Extract and Fractions on Phagocytic Activity of Mouse Peritoneal Macrophages

**Figure 4:**
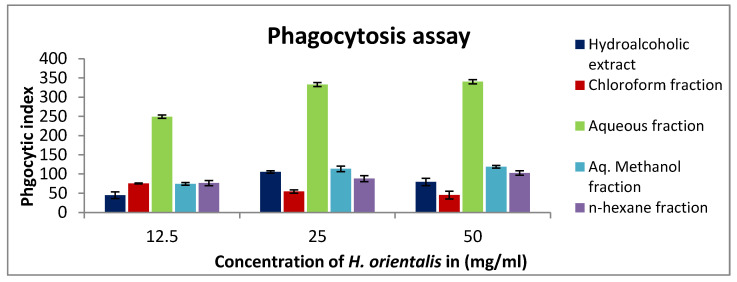
In vitro phagocytic assay using nitro blue tetrazolium (NBT) reduction test of peritoneal macrophages treated with various concentrations (12.5–50 mg/mL) of *H. orientalis* L. extract and fractions. Aqueous fraction had the highest phagocytic index (340). Results are expressed as means (bars) ± SEM (lines).

A concentration-dependent enhancement of phagocytic activity was observed in macrophages treated with 12.5–50 mg/mL *H. orientalis* L. (Figure 4). At a concentration of 50 mg/mL, the aqueous fraction showed the highest stimulation of peritoneal phagocytic activity compared to the negative control, followed by aqueous methanol fraction.

### 2.6. Effect of H. orientalis L. Extract and Fractions on Pinocytic Activity of Mouse Peritoneal Macrophages

**Figure 5:**
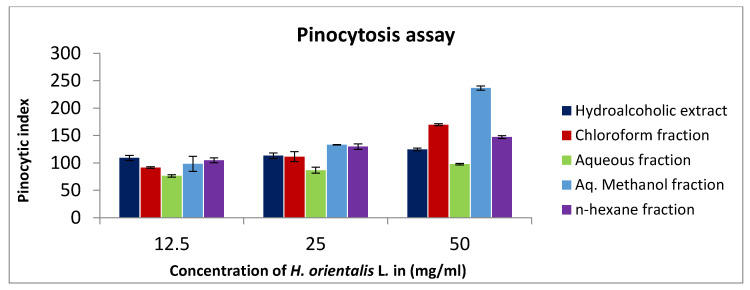
In vitro pinocytic assay using neutral red method on peritoneal macrophages treated with different concentrations (12.5–50 mg/mL) of *H. orientalis* L. extract and fractions. Aq. Methanol showed the highest pinocytic index (236). Results are expressed as means of three independent experiments (bars) ± SEM (lines).

Neutral red method was used to demonstrate the pinocytic activity after treatment with different *H. orientalis* L. extract and fractions. At concentration of 50 mg/mL, aqueous methanol fraction showed the highest activity with pinocytic index value of 236 (Figure 5). On the other hand, other fractions were less active compared to the negative control.

### 2.7. Effect of H. orientalis L. Extract and Fractions on Cytokines Level

**Figure 6:**
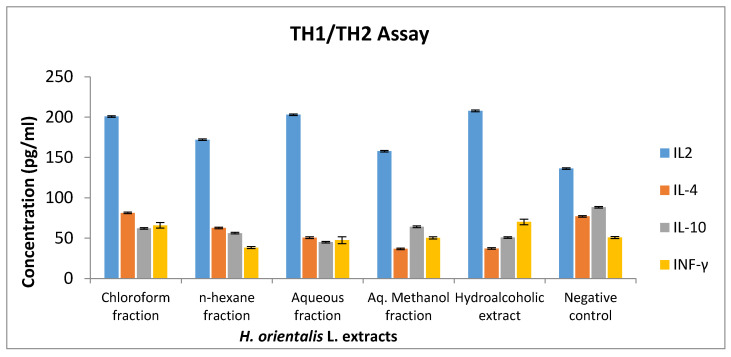
The effect of *H. orientalis* L. extract and fractions on IL-2, IL-4, IL-10, and IFN-γ level at concentration of 50 mg/mL. TH1/THI murine assay kit was used to measure the effect of the *H. orientalis* on cytokines. Secretion of IL-2 was enhanced in the presence of hydroalcoholic extract and chloroform fraction. Results are expressed as means of three independent experiments (bars) ± SEM (lines).

The results demonstrated that there is an increase in IL-2 levels in lymphocytes treated with 50 mg/mL of various extract and solvent fractions compared to the negative control (Figure 6). Hydroalcoholic extract and chloroform fraction induced the highest increase in IL-2, as well as IFN-γ.

### 2.8. Total Phenolic Content of H. orientalis L. Polar Extract and Fractions

**Figure 7:**
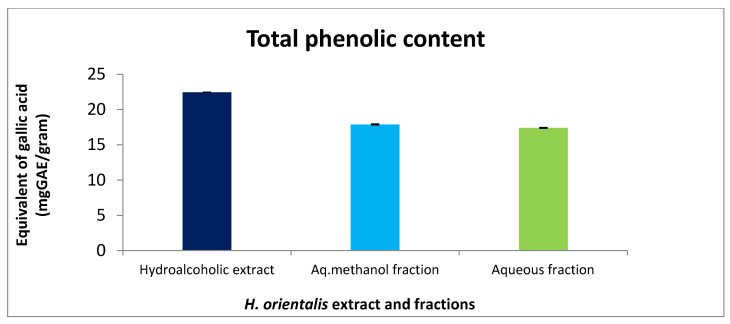
Total phenolic content in mg GAE/g dry weight of *H. orientalis* L. polar extract and fractions at concentration 100 mg/mL. Hydroalcoholic extract showed the highest value (22.4 mg GAE/g dry weight). Results are expressed as means of three independent experiment (bars) ± SEM (lines).

Foline–Ciocalteu assay was applied to detect the content of phenol in *H. orientalis* L. extracts. Hydroalcoholic extract and aq. methanol fraction showed the highest equivalent of gallic acid with values of 22.4 and 17.8 mg GAE/g dry weight, respectively (Figure 7).

### 2.9. LC–MS Analysis of H. orientalis L. Extract and Fractions

LC–MS analysis showed that trans ferulic acid presented in high percentage in each of *n*-hexne, chloroform fractions, and hydroalcoholic extract with values of 47.1%, 33.3%, and 33.2%, respectively. On the other hand, rosmarinic acid was detected abundantly in aqueous fraction with percentage value of 78.6%. Additionally, p-coumaric acid was found in 30.3% in *n*-hexane fraction (Table 3). These percentages are relative values depending on the compounds detected in the extract and fractions (Figures S1–S5).

### 2.10. Peganine and Sabinene Are the Main Components of H. orientalis L. Hydroalcoholic Extract

Analysis of the hydroalcoholic extract using GC–MS/MS revealed the presence of a high concentration of peganine and sabinene with percentages of 25.4% and 19.3%, respectively (Table 4). Some other compounds found in different concentrations were limonene (12.3%), ruine (9.5%), linalool (7.1%), myrtenal (6.9%), and alpha-pinene (3.5%).

### 2.11. Toxicity Evaluation of H. orientalis L. Hydroalcoholic Extract

Based on the mortality percentage of the pilot study, the dose of 4000 mg/kg was selected to be the base dose for LD₅₀ determination assay. The LD₅₀ was determined according to the arithmetical method of Karber (Table 5). All mice in group five died, while only two mice died in group four. No death was observed in groups three and two. The LD₅₀ value of the *H. orientalis* hydroalcoholic extract was 3000 mg/kg, and as a result the preliminary therapeutic dose for the in vivo study was 1/10 of the LD₅₀ value.

### 2.12. Antitumor Effects of H. orientalis L. Hydroalcoholic Extract on EMT6/P Cells Implanted in Mice

**Figure 8:**
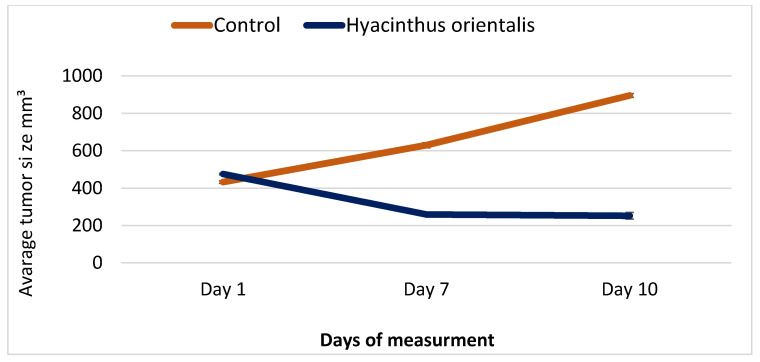
A plot demonstrated the changes in average tumor size (mm³) vs. time in (days) of treatment with *H. orientalis* L. hydroalcoholic extract in EMT6/P cell line.

**Figure 9:**
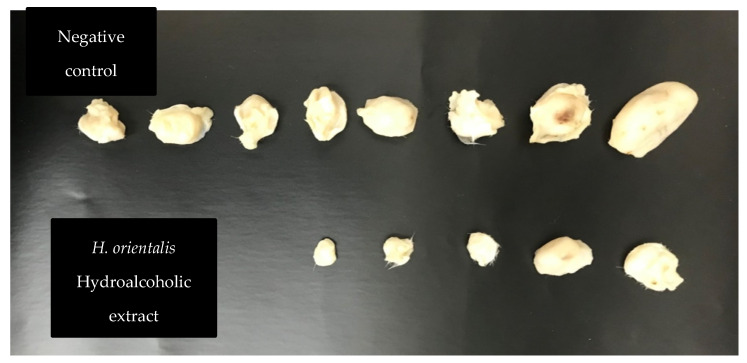
Effect of *H. orientalis* L. hydroalcoholic extract on tumor size and cure percentage. Treatment with *H. orientalis* resulted in smaller tumors size and higher cure percentage compared to the negative control (*n* = 10 mice in each group).

Hydroalcoholic extract was selected to treat tumor bearing mice, as it showed the highest activity in the in vitro experiments. Treatment of tumor-bearing mice with *H. orientalis* L. hydroalcoholic extract showed significant (*p* < 0.05) reduction of tumor size (−46.94%) compared to the negative control (107.01%) (Table 6 and Figure 8; Figure 9). The percentage of mice with no detectable tumor in hydroalcoholic extract-treated group was 50%. In addition, mice exhibited normal activity with no side effects.

## 3. Discussion

*H. orientalis* L. is widely used in traditional medicine to treat different ailments. The plant was traditionally used for prostate disease, wound healing, and hemorrhoids. Whole plant leaves and bulbs were tested in different traditional recipes [18]. In the current study, the anticancer and immunomodulatory effects of *H. orientalis* L. extracts were evaluated. The results revealed that the plant exhibits high potential to stimulate the immune system and inhibit cancer growth.

In the antiproliferative assay, all extracts showed various degrees of activity against cancer cell lines with limited toxicity toward Vero cells. The highest activity was reported for the aqueous methanol fraction which inhibited cancer cell with concentrations below 0.25 mg/mL and showed limited toxicity against EMT6/P and Vero cell lines. However, other solvent fractions were also effective at various concentrations against some cell lines.

For better understanding of the mechanism of action of these extract and fractions, apoptosis (programmed cell death) induction was measured using caspase-3 activity assay. Apoptosis induction is a complex process that can be achieved by two mechanisms: intrinsic (mitochondrial-dependent) and extrinsic (death receptor-dependent) [19]. The release of cytochrome c from mitochondria is the starting signal for intrinsic pathway [20], while binding of ligand to death receptor is the starting signal for extrinsic pathway [21]. Both pathways end by the activation of caspases and cell death. Caspase-3 is an executioner caspase activated in both pathways [19], and measurement of its activity after treatment with extracts is an indication of apoptosis induction. Apoptosis inhibition is one of the main mechanisms used by cancer cells to secure survival. Thus, induction of apoptosis was suggested as an effective mechanism to inhibit cancer [22]. Therefore, the extracts were tested at their IC_50_ values for their apoptotic effect. The results of this study have shown that the hydroalcoholic extract had the highest activity. Enhanced caspase-3 activity was three times higher than the negative control. Other solvent fractions showed moderate to low apoptosis-inducing effects. The observed apoptosis could be explained by the presence of high percentage (25.4%) of pegamine in hydroalcoholic extract. This compound was reported to have apoptosis-induction ability by inhibiting the enzyme topoisomerase I [23]. Another compound that may have participated in apoptosis induction is limonene, which was also detected in high percentage (12.3%) in the extract. Earlier study has shown that limonene induces apoptosis by inhibiting PI3K/Akt pathway [24]. Moreover, rosmarinic acid was detected in high concentration in different extracts of *H. orientalis* L. It was reported that rosmarinic acid has the ability to promote cell arrest in the G₀/G₁ phase and modulate the expression of many apoptosis-involved genes [25]. Additionally, trans-ferulic acid was found at various percentages in four different extracts of *H. orientalis* L. This compound has shown antiproliferative activity as well as induced the expression of pro-apoptotic factor Bax, and reduced the expression of pro-survival factor survivin [26].

Phenolic compounds exhibited anticancer activity. It affects multiple key elements related to the cell proliferation, apoptosis, angiogenesis, and metastasis [27]. Hydroalcoholic extract and aq. methanol fraction showed high phenolic content compared to the other fractions.

Human immune system is composed of sophisticated network of cells and organs that work together to provide protection against different pathogens [28]. In our study, we evaluated the change in the function of innate and acquired immune response after treatment with different extract and fractions from *H. orientalis* L. In the lymphocyte proliferation assay, the highest response was observed in *n*-hexane fraction followed by hydroalcoholic extract. Both activated lymphocyte proliferation in the presence and absence of mitogens (lipopolysaccharide (LPS) and concanavalin A (Con-A)).

Acquired immune response can be shifted toward T helper-1 (Th1) or T helper-2 (Th2) response depending on the type of cytokines produced. IL-4 is the key cytokine for Th1 response, while IL-2 and INF-γ is the signature cytokine for Th1 [29]. A balanced ratio of Th1/Th2 cytokines was observed in healthy individuals. However, a shift in the immune response toward Th2 was reported in cancer patients [30]. An activation of Th1 immune response is associated with anticancer immune response [31]. Our results revealed an increase in the level of IL-2 and INF-γ in lymphocytes treated with the hydroalcoholic extract. These results indicate a shift in the immune response toward Th1 antitumor response, which may explain the potent antitumor effect of this extract.

Evaluating the innate immune response showed an effect of this plant in activating innate immunity. An enhanced macrophage phagocytic activity was observed after treating the cells with increasing concentrations of different fractions. The highest activity was reported for the *n*-hexane fraction. On the other hand, aqueous methanol fraction showed the highest potential in activating pinocytosis. Both processes (phagocytosis and pinocytosis) are essential for initiating the antitumor immune response by involving in antigen presentation to activate T-lymphocytes [32]. The immune stimulating properties of *H. orientalis* L. could be the result of specific active compounds present in this plant. Earlier study has shown that limonene increases phagocytosis in tumor bearing mice [33]. However, when used at a high concentration, it was shown to reduce proliferation of lymphocytes [34]. It seems that the lymphocytes proliferating effect of *H. orientalis* L. is due to the presence of other components in this plant and not due to limonene. Compounds like saponin, quinone, and rosmarinic acid detected in this plant may have contributed to the immunomodulatory response by modulating lymphocytes function [35,36,37].

In the current study, the hydroalcoholic extract of *H. orientalis* L. was used to treat mice implanted with breast cancer. Significant reduction in tumor size along with high cure percentage (50%) were reported. Such regression in tumor size could be a result of the presence of effective anticancer agents like peganine, limonene, alpha-pinene, saponin, and quinone. These compounds may work synergistically to inhibit cancer by activating the immune system and stimulating apoptosis.

Overall, results of this study revealed a promising anticancer effect of extracts obtained from *H. orientalis* L. Phytochemicals in this plant work in different anticancer mechanisms including inhibition of cell proliferation, induction of apoptosis, and modulation of the acquired and innate immune responses. The study can be further expanded to clearly understand the mechanisms of action of these extracts and to develop potential anticancer therapies based on natural products.

## 4. Materials and Methods

### 4.1. Animals

Balb/C female mice (4–6-week-old, weight 21–25 g) were used in this study. The environmental conditions in the animal room were: 50–60% humidity, 25 °C with continuous air ventilation, and alternating light/dark cycles of 12 h. All animal experiments were evaluated and approved by the Research and Ethical Committee of Applied Science Private University (Approval Number: 2015-PHA-05). The total number of mice used in this study was 54 (Table 7).

### 4.2. Cell lines and Cell Culturing Conditions

Two human epithelial breast cancer cell lines (T47D and MCF-7), mouse mammary cell line (EMT6/P), and Kidney epithelial cells from African green monkey (Vero) were utilized to examine the impact of *H. orientalis* L. extracts against their survival. All cell lines were provided from The European Collection of Authenticated Cell Cultures (ECACC) (Uk). The Vero cell line was used as a model of normal cells to measure the toxicity of extracts in vitro. EMT6/P cells were used to induce breast cancer in mice. These cells were cultured in the complete medium and incubated at 37 °C in 5% CO_2_ and 95% humidity incubator. T47D and MCF-7 cell lines were cultured in complete RPMI 1640 medium, while EMT6/P was cultured in complete minimum essential medium. Kidney epithelial cells were applied as normal control and cultured in complete DMEM medium. All culture media were supplied with 1% L-glutamine, 10% fetal bovine serum, 1% penicillin-streptomycin, and 0.1% gentamycin solution.

### 4.3. Plant Collection, Moisture Content, and Extracts Preparation

**Figure 10:**
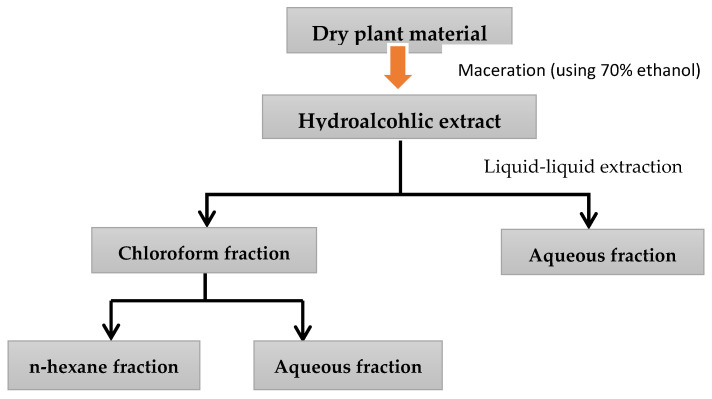
Steps of extract and fractions preparation.

*H. orientalis* L. was purchased from the United Arab Emirates market. The taxonomic identity of the plant was authenticated by botanists in the royal society for the conservation of nature, Amman, Jordan. Fresh plant was dried at 30 °C with ventilation, powdered mechanically, and stored in well-closed glass containers in a dry–dark place. Moisture content was determined using oven-drying method [38]. Briefly, powdered plant material was weighed and dried in oven at 135 °C for 2 h. The weight was measured again after drying to calculate the moisture content. Different extracts were prepared by different solvents. Hydroalcoholic extract was prepared by macerating the plant dried powder in 70% ethanol (1 L per 100g) in a lab mixer for three days. After that, the extract was filtered, and the supernatant was concentrated using rotary evaporator. Complete drying of the extract was performed using lyophilizer, and then the extract was kept at −20 °C until used. Water, aqueous methanol, chloroform, and *n*-hexane fractions were prepared from hydroalcoholic extract using liquid–liquid fractionation method. These solvents were selected as they help to separate phytochemicals based on their polarity. Hydroalcoholic extract was subjected to solvent–solvent partitioning between chloroform and water. One hundred grams of hydroalcoholic extract was mixed with 100 mL chloroform followed by adding 100 mL water with continuous mixing. The two solvents were loaded in a separating funnel (250 mL volume) and left to separate overnight. Each solvent was collected in a clean beaker and the solvents were evaporated using rotary evaporator. After drying both fractions, the chloroform fraction was also subjected to solvent–solvent partitioning between *n*-hexane and aqueous methanol (Figure 10). All the fractions were dried completely and stored at −20 °C until used [39]. Figure 10 summarizes the extraction preparation and solvent—solvent partitioning.

### 4.4. Antiproliferative Assay

Different cell lines were distributed (100 μL/well) into 96-well tissue culture plates (flat bottom) and seeded at 15,000 cells/well in complete tissue culture medium. This cell density was selected as it gives confluence layer after 48 h incubation for the cell lines used in this study. After 24 h, the media in each well were removed and then the attached cells were treated in triplicate with *H. orientalis* L. different extracts (initially dissolved in 0.1% DMSO). Using serial dilution technique, the extract and fractions were added in concentrations between 50–0.78 mg/mL and re-incubated for 48 h. Cell viability was measured using MTT [3-(4,5-Dimethylthiazol-2-yl)-2,5-diphenyltetrazolium bromide] assay kit (Sigma, USA) as previously described [40]. Briefly, after 48 h incubation with 100 μL of extracts and fractions, media was removed from each well and 10 μL MTT was added. Cells were incubated with MTT for 3 h followed by adding stop solution. One hour after adding the stop solution, the developed color was measured using microplate reader. Percentage survival was estimated by comparing the absorbance of treated cells to the negative control (tissue culture media and 0.1% DMSO). The half maximal inhibitory concentration (IC₅₀) was calculated using non-linear regression in SPSS. The assay was conducted on three breast cancer cell lines (MCF-7, T47D, and EMT6/P) and one normal cell line (Vero). Vero cell line was used to determine the toxicity of extracts on normal cells.

### 4.5. Apoptosis Detection in T47D Cells

T47D cells were cultured in six separated 25 cm² flasks at a concentration of 150,000 cells/mL of complete tissue culture medium. After 24 h of incubation, the media in each flask was discarded and the attached cells were treated with *H. orientalis* L. different extract and fractions at the IC_50_ concentration of each extract. Concentrations of the *H. orientalis* L were as the following: hydroalcoholic (6 mg/mL), chloroform (3.5 mg/mL), aqueous (11 mg/mL), aqueous methanol (0.1 mg/mL), and *n*-hexane (0.6 mg/mL). Following incubation for 48 h, media of each flask was removed, and the attached cells were harvested. Caspase-3 activity was measured according to the kit instructions (caspase-3 assay kit, Abcam, Cambridge, MA, USA). Briefly, cells were mixed with chilled cell lysis buffer for 10 min followed by centrifugation at 10,000× *g* for 1 min. Supernatants were aspirated and DEVD-p-nitroanilide was added to the supernatants as a substrate and incubated for two hours. Color development was measured at 405 nm using spectrophotometer. Fold-increase in caspase-3 activity was detected by comparing extracts results with the level of the negative control. Procedure of the assay was carried out as described previously [40].

### 4.6. Preparation of Murine Splenocytes

Three Balb/C mice were used in this experiment. Mice were sacrificed, and the spleen was withdrawn under aseptic conditions from each mouse. Spleen cells were moved through a mesh of a tissue grinder, and the cell suspension was prepared in complete RPMI-1640 media. Then the cell suspension was washed thrice for 10 min utilizing complete RPMI 1640 media and subsequently re-suspended in 5 mL red blood cells lysis buffer (1 mol/L NH4Cl). Following 10 min, the cells were centrifuged again and re-suspended in complete RPMI-1640 media. Splenocytes were washed and cell viability was determined using trypan blue assay.

### 4.7. Lymphocytes Proliferation Assay

This assay was performed using MTT -based colorimetric assay according to previously described method [41]. Concisely, splenocytes suspension was made in complete RPMI-1640 solution at the concentration of 2 × 10^6^ cell/mL, and then seeded into 96-well culture plate in the presence of mitogens. Con A (concanavalin A) (5 μg/mL) and LPS (lipopolysaccharide) (4 μg/mL) were used as mitogens for T and B lymphocyte, respectively. To each well, 100 μL of different concentrations of *H. orientalis* L. extract and fractions (12.5–50 mg/mL) were added (triplicate). Moreover, the negative control contains the same volume of RPMI-1640 medium. The plate was incubated for 48 h under 5% CO_2_ and a humidified atmosphere at 37 °C temperature. Following the 48 h of incubation, 15 μL MTT solution was added to each well. Then, microplate was incubated for 4 h. After that, 100 μL DMSO was added per well to dissolve the formazan crystals, and the absorbance was measured at 570 nm using ELISA microplate absorbance reader. Results were demonstrated as a percentage of proliferation (%) compared to the negative control cells. The same procedure was repeated with the exclusion of the addition of Con A and LPS [42].

### 4.8. Macrophage Isolation from Peritoneal Fluid

Three Balb/C mice were used in this experiment. Peritoneal macrophages (PEM) were isolated from peritoneal cavity as mentioned previously [43]. Concisely, PEM were harvested from 5 Balb/C mice, which were already injected intraperitoneally with 3 mL of thioglycollate three days before getting euthanized by cervical dislocation. Then, 5 mL ice-cold PBS was introduced into the mouse cavity. Mild massaging was applied, and then fluid was collected and placed in conical tubes carried on ice. Following centrifugation of the pooled fluid (1000 rpm, 10 min, 4 °C), the resulting pellet of the cells was suspended in complete RPMI 1640 medium at a density of 2 × 10^6^ cells/well and permitted to adhere for 3 h at 37 °C in 5% CO_2_ humidified incubator. After that, non-adherent cells were washed away with RPMI 1640 medium, then collected for use in different assays outlined below [44].

### 4.9. In Vitro Phagocytic Assay (Nitro Blue Tetrazolium (NBT) Reduction Test)

The NBT reduction assay was conducted according to the process of Rainard [45]. Briefly, PEM (5 × 10^6^ cells/well) were cultured in 96-well plate with different concentrations of *H. orientalis* L. extracts (12.5–50 mg/mL) for 48 h at 37 °C in the incubator. Consequently, 20 μL yeast suspension (5 × 10⁶ cells/well in PBS) and 20 μL nitro blue tetrazolium (NBT) (1.5 mg/mL in PBS) were added to each well. Negative controls wells received 20 µl PBS and 20 µl DMSO. The plate was incubated for 60 min at 37 °C. Afterward, the supernatant was discarded, and the adherent macrophages were washed with RPMI 1640. The cells were air-dried before 120 μL of 2M KOH and 140 μL DMSO were added to each well. The absorbance was measured at 570 nm in the microplate reader. Phagocytic activity was calculated based on the following equation [44]:

Stimulation index = (OD sample − OD control)/OD control * 100

### 4.10. Determination of Pinocytic Activity Using Neutral Red Method

Peritoneal mice macrophages were seeded into a 96-well plate. *H. orientalis* L. extract and fractions were added in three different concentrations (50, 25, and 12.5 mg/mL) and incubated for 48 h at 37 °C in the incubator. Neutral red solution was prepared (7.5 mg/mL in PBS), and then added (100 μL) to each well. The plate was incubated for 2 h. Supernatant was aspirated and the wells were washed with PBS many times to remove the remaining particles of the neutral red. Cells were disintegrated using cell lysis solution (ethanol and 0.01% acetic acid at the ratio 1:1), as 100 μL was added to each well and overnight incubated at room temperature. Microplate reader was used to read the absorbance at 540 nm. Pinocytic activity was expressed as pinocytic index and calculated as described in the phagocytosis section [45].

### 4.11. Effect of H. orientalis L. Extracts on Cytokines Levels in Activated Lymphocytes

Murine splenocytes (2 × 10⁶ cells/mL) were cultured with various extracts of *H. orientalis* L. (50 mg/mL) for 48 h in CO₂ incubator at 37 °C. Levels of IFN-γ, IL-2, IL-4, and IL-10 were measured using TH1/TH2 ELISA kit (affymetrix ebioscience, Canada). After period of incubation, supernatants were collected and used to estimate the level of cytokines according to the standard kit procedure [46]. Based on the calculated standard curve of each cytokine, absorbance values were converted into concentration (pg/mL).

### 4.12. Detection of Total Phenolic Content (TPC) Using Folin–Ciocalteu Method

A total content of phenol in *H. orientalis* L. polar extract and fractiona was determined according to the procedure demonstrated by Akyuz with some modifications [47]. Samples were prepared at concentration of 100 mg/mL. In a 96-well plate (flat bottom), 12.5 μL from the *H. orientalis* L. was added to each well in triplicate, followed by 250 μL of 2% sodium carbonate solution. They were left for 5 min at room temperature (RT). Then, 12.5 μL of 50% of Folin–Ciocalteu reagent was added and left for 30 min at RT. Microplate reader was used to record the absorbance of the mixture at 630 nm. The phenolic content was estimated as gallic acid equivalents GAE/g of dry weight on the basis of a standard curve of gallic acid. The standard curve was prepared using gallic acid solution at different concentrations (0.1–1 mg/mL) in distilled water.

### 4.13. LC–MS Measurements of H. orientalis L. Extracts

Samples have been prepared by dissolving them in 2 mL DMSO (dimethyl sulfoxide) and completed the volume to 50 mL with acetonitrile. Centrifuge of each sample was conducted at 4000 rpm for 2 min, then 1 mL has been transferred to the autosampler. Injection volume was 3 μL. The analysis was performed using Burker Daltonik (Berman, Germany) impact Ⅱ ESI-Q-TOF system equipped with Burker Dalotonik Elute UPLC system (Beremen, Germany). The instrument was operated using the Ion Source Apollo Ⅱ ion funnel electrospray source (capillary voltage: 2500 v; nebulizer gas: 2 bar; dry gas flow: 8 L/min; dry temperature: 200 °C; mass accuracy: less than 1ppm; mass resolution: 50,000 FRS; the TOF repetition rate: 20 kHz). Chromatographic separation was performed using Burker solo 2-C-18 UHPLC column (100 mm × 2.1 mm × 2 μm) at flow rate of 0.51 mL/min and a column temperature of 40 °C. All standards were used for identification of ms/z and the retention time.

### 4.14. GC–MS Analysis of H. orientalis L. Hydroalcoholic Extract

Analysis was performed by using GC-2010 plus gas chromatography (Shimadzu, Tokyo, Japan) coupled to a GCMS-QP 2010 SE mass spectrometry detector (Shimadzu, Tokyo, Japan) and equipped with an AOC20i auto-injector (Shimadzu, Tokyo, Japan). A capillary Rtx-5MS column (30 m × 0.25 mm i.d × 0.25 μm film thickness, Restek, Bellefonte, PA, USA) was used for separation. Helium (at flow rate of 1.0 mL/min) was used as carrier gas. Temperature was kept at 60 °C for 5 min and set to reach 240 °C at the rate of 3 °C per min. The samples were injected at a temperature of 250 °C. The injection volume was 1.0 μL in 1:30 split ratio. The mass spectra were acquired with electron impact ionization (70 Ev) at full scan mode (40 to 500 *m/z*), utilizing an ion source at 200 °C. The detection of compounds was achieved using NIST (National Institute of Standards and Technology, Gaithersburg, MD, USA) mass spectral library.

### 4.15. Acute Toxicity of H. orientalis L. Hydroalcoholic Extract

This procedure was conducted to determine the toxicity of the extract (hydroalcoholic extract) on normal mice, which will help to determine the therapeutic dose. A pilot study was applied on a small group of mice to choose the dose ranges for actual LD₅₀ (median lethal dose) determination. Briefly, the hydroalcoholic extract was dissolved in PBS containing 5% of tween 20. Two female mice (six weeks old, 20–23 g weight) were injected intraperitoneally with a specific dose of the extract, followed by observation of mortality for 24 h. The next doses were increased by 1.5 if the dose was tolerated, or reduced by 0.7 if it was lethal using new animals. The maximum non-lethal and minimum lethal doses represented the lower and upper limits, which were used to prepare LD₅₀ doses [48]. For LD₅₀ determination, four groups (*n* = 4) of mice were injected intraperitoneally with different concentrations (2500, 3000, 4000, and 5000 mg/kg) of the extract within the higher and lower range detected in the pilot study. The untreated fifth group (*n* = 4) was used as a negative control and injected IP with PBS and 5% tween 20. Mice were observed for 24 h for mortality and general behavior. The LD₅₀ was indicated by the concentration that showed 50% mortality applying the arithmetical method of Karber [20].

### 4.16. Antitumor Activity on Experimental Animals

The mouse mammary tumor cells EMT6/P were harvested by trypsinization and tested for their viability using trypan blue exclusion method. Twenty Balb/C mice were injected subcutaneously in the abdominal area with tumor induction dose of 1 × 10^5^ cells in 0.1 mL. After 14 days, the dimensions of the grown tumors were measured using digital caliper. Then, tumor volumes were measured according to the following formula: (A × B² × 0.5), where A was the length of the longest aspect of the tumor and B was the length of the perpendicular to A [49]. Tumor-bearing mice were set into two groups each with 10 mice. Group 1 represented the negative control and received daily intraperitoneal injection (100 μL) of the vehicle (5% tween 20 in PBS). Group 2 was treated with hydroalcoholic extract of *H. orientalis* L. at a dose of 300 mg/kg (10% of the determined LD₅₀). The hydroalcoholic extract was selected for the in vivo antitumor assay, as this extract represents the main extract used in the first step in the extraction procedure, and all other fractions were fractionated from hydroalcoholic extract. Additionally, the hydroalcoholic extract showed moderate to high activity in all in vitro anticancer and immunomodulatory assays. All treatments were pursued for 10 days, and then tumors were remeasured before mice were sacrificed (using cervical dislocation). Tumors were removed and stored in 10% formalin.

### 4.17. Statistical Analysis

Quantitative data were shown as mean ± standard error. The statistical significance among the groups was carried out by using a one-way analysis of variance (ANOVA) and student’s *t*-test. A *p*-value < 0.05 was considered significant. The IC₅₀ values obtained with the different concentrations of plant extract were calculated using nonlinear regression in SPSS (Statistical Package for the Social Sciences, Chicago, IL, USA, version 24).

## 5. Conclusions

*H. orientalis* L. is a plant with high anticancer and immunomodulatory effects. The anticancer activity of this plant is mediated by apoptosis induction, while the immunomodulatory effect involves activation of innate and acquired immune responses. Both effects are the result of the presence of biologically active compounds in the plant extracts. Therefore, *H. orientalis* L. can be considered a source of anticancer and immunomodulatory agents. However, further testing is needed to investigate the chemical composition of this plant and understand its mechanisms of action, including the ERK or PI3K-Akt/PKB signaling pathways.

## Figures and Tables

**Table 1:** The percentage yield obtained from the extraction of 2000 gm of *H. orientalis* using maceration and solvent fractionation methods (% yield = weight after extraction/weight before extraction * 100%).

Source	Extraction Method	Extraction Solvent	% of Dried Extracts Yields
*H. orientalis*	Maceration	Hydroalcoholic (ethanol 70%)	24.3%
	Fractionation	Chloroform	20.7%
		Water	46%
		Aqueous/Methanol	6.6%
		*n*-Hexane	12%

**Table 2:** IC₅₀ values of *H. orientalis* L. extract and fraction against different cell lines.

*H. orientalis* L. Extracts	MCF-7 Cell Line	T47D Cell Line	EMT6/P Cell Line	VERO Cell Line
Hydroalcoholic extract IC₅₀ (mg/mL)	0.95 ± 0.07	6.12 ± 0.1	3.65 ± 1.2	>50
Chloroform fraction IC₅₀ (mg/mL)	0.70 ± 0.2	3.53 ± 0.5	1.53 ± 0.2	>50
Aqueous fraction IC₅₀ (mg/mL)	6.59 ± 0.01	11.38 ± 1.2	11.78 ± 0.6	>50
n-hexane fraction IC₅₀ (mg/mL)	2.23 ± 1.5	0.59 ± 0.02	3.65 ± 1.2	>50
Aq. Methanol fraction IC₅₀ (mg/mL)	0.24 ± 0.04	0.11 ± 0.08	3.06 ± 0.5	>50

**Table 3:** LC–MS analysis results of *H. orientalis* L. extract and fractions.

NO	Compounds	Formula	RT	Relative % (Hydroalcoholic Extract)	Relative % (Chloroform Fraction)	Relative % (Aq. Methanol Fraction)	Relative % (*n*-hexane Fraction)	Relative % (Aqueous Fraction)
1	Caffeic Acid	C9H8O4	3.31	0.35%	0.60%	0.44%	0.00%	1.47%
2	Vanillin	C8H8O3	3.75	3.16%	1.87%	2.34%	3.18%	0.44%
3	p-Coumaric acid	C9H8O3	4.44	22.74%	18.85%	21.08%	30.31%	0.44%
4	4’-O-GlcA-7-O-GlcA Apigenin (NMR)	C27H26O17	4.66	0.00%	0.01%	0.01%	0.00%	0.16%
5	Ferulic acid (trans)	C10H10O4	5.21	33.21%	33.37%	30.13%	47.17%	0.87%
6	2,4-Dihydroxyacetophenone	C8H8O3	5.21	1.78%	2.16%	2.36%	3.00%	0.04%
7	3-Hydroxy-4-methoxycinnamic acid (isoferulic acid)	C10H10O4	5.66	0.06%	0.04%	0.06%	0.00%	0.28%
8	Luteolin 7-O-glucoside (Cynaroside)	C21H20O11	5.92	3.83%	2.58%	3.37%	5.98%	0.01%
9	Apigenin-7-O-glucoside (Apigetrin)	C21H20O10	6.81	4.46%	2.42%	3.63%	5.42%	0.06%
10	Rosmarinic acid	C18H16O8	7	17.99%	17.30%	23.58%	0.15%	78.67%
11	(4 or 7) Hydroxy-Coumarin Plus Hydrate	C9H6O3	7	2.41%	2.76%	3.99%	0.00%	9.84%
12	3,7,4’-Trihydroxyflavone (5-Deoxykampferol)	C15H10O5	8	0.13%	0.03%	0.09%	0.00%	0.51%
13	3,6,2’,3’-Tetrahydroxyflavone	C15H10O6	8.61	1.63%	2.54%	2.11%	0.13%	3.95%
14	Baicalein	C15H10O5	9.92	2.21%	5.83%	2.61%	0.45%	2.38%
15	Apigenin	C15H10O5	9.93	2.47%	6.09%	3.43%	1.59%	0.97%
16	Galangustin	C17H14O6	10.6	0.10%	0.23%	0.16%	0.01%	0.32%
17	9Z, 11E-Linoleic acid	C18H32O2	29.77	1.49%	0.00%	0.00%	2.60%	0.00%
18	Linoelaidic acid	C18H32O2	30.07	0.00%	3.33%	0.00%	0.00%	0.00%
19	Stearic acid	C18H36O2	32.18	1.99%	0.00%	0.61%	0.00%	0.00%

**Table 4:** Major compounds identified in the *H. orientalis* L. hydroalcoholic extract using GC–MS method.

No	Compound	Formula	Molecular Weight (MW)	RT	%
1	alpha-pinene	C10H16	136	9.92	3.5
2	Ruine	C19H22N2O7	390	11.22	9.5
3	Sabinene	C10H16	136	14.83	19.3
4	Peganine	C11H12N2O	188	17.45	25.4
5	Limonene	C10H16	134	19.92	12.3
6	Camphor	C10H16O	152	32.5	6.7
7	Linalool	C10H18O	154	34.99	7.1
8	Myrtenal	C10H14O	150	37.72	6.9
9	Saponin	C97H137NO17Si2	1645	40.02	2.95
10	Quinone	C6H4O2	108	42.61	2.11

GC–MS: Gas chromatography coupled to the tandem mass spectrometry; MW: molecular weight; RT: retention time.

**Table 5:** Acute toxicity assay of *H. orientalis* L. hydroalcoholic extract in mice (*n* = 4) injected intraperitoneally and observed for 24 h. *n* = number of mice in each group.

Groups (*n* = 4)	Dose (mg/kg)	No. of Mortality	Dose Difference (a)	Mean Mortality (b)	Probit (a × b)
1	PBS + 5% tween 20	0	0	0	0
2	2500	0	0	0	0
3	3000	0	500	0	0
4	4000	2	1000	1	1000
5	5000	4	1000	3	3000

Dose difference (a) = higher dose – lower dose. Mean mortality (b) = (mortality in the second concentration + mortality in the first concentration)/2.

**Table 6:** Effect of *H. orientalis* L. hydroalcoholic extract on tumor size and cure percentage.

Treatment Groups (*n* = 10)	Initial Tumor Size (mm³) ± SEM	Final Tumor Size (mm³) ± SEM	% Change in Tumor Size	% of Mice with no Detectable Tumor	Average Tumor Weight (g)
Control	432.62 ± 11.1	895.61 ± 35.4	107.01	20%	0.71
*H. orientalis* hydroalcoholic extract	475.25 ± 18.4	252.13 ± 21.3	-46.94	50%	0.31

*n* = 10, mm³: cubic millimeter.

**Table 7:** The distribution of mice on different experiments.

No. of Mice	Assessment Study	No. of Mice	Assessment Study
8	Pilot study	20	Anti-tumor assay
20	Acute toxicity assay	6	Immune assay

## Data Availability

Not applicable.

## Supplementary Materials

The following are available online at https://www.mdpi.com/2223-7747/10/4/617/s1, Figures S1–S5. LC-MS chromatograms of *H. orientalis* hydroalcohlic extract and fractions.

## References

[1] Bray F., Ferlay J., Soerjomataram I., Siegel R.L., Torre L.A., Jemal A. (2018). Global cancer statistics 2018: GLOBOCAN estimates of incidence and mortality worldwide for 36 cancers in 185 countries. CA Cancer J. Clin..

[2] Housman G., Byler S., Heerboth S., Lapinska K., Longacre M., Snyder N., Sarkar S. (2014). Drug resistance in cancer: An overview. Cancers.

[3] Kuruppu A.I., Paranagama P., Goonasekara C.L. (2019). Medicinal plants commonly used against cancer in traditional medicine formulae in Sri Lanka. Saudi Pharm. J. SPJ Off. Publ. Saudi Pharm. Soc..

[4] Graham J.G., Quinn M.L., Fabricant D.S., Farnsworth N.R. (2000). Plants used against cancer—An extension of the work of Jonathan Hartwell. J. Ethnopharmacol..

[5] Khan T., Ali M., Khan A., Nisar P., Jan S.A., Afridi S., Shinwari Z.K. (2019). Anticancer Plants: A Review of the Active Phytochemicals, Applications in Animal Models, and Regulatory Aspects. Biomolecules.

[6] Rayan A., Raiyn J., Falah M. (2017). Nature is the best source of anticancer drugs: Indexing natural products for their anticancer bioactivity. PLoS ONE.

[7] Leng J.C., Gany F. (2014). Traditional Chinese medicine use among Chinese immigrant cancer patients. J. Cancer Educ..

[8] Damery S., Gratus C., Grieve R., Warmington S., Jones J., Routledge P., Greenfield S., Dowswell G., Sherriff J., Wilson S. (2011). The use of herbal medicines by people with cancer: A cross-sectional survey. Br. J. Cancer.

[9] Christopher B. (1996). The Royal Horticultural Society AZ Encyclopedia of Garden Plants.

[10] Hu F., Liu H., Wang F., Bao R., Liu G. (2015). Root tip chromosome karyotype analysis of hyacinth cultivars. Genet. Mol. Res..

[11] Altundag E., Ozturk M. (2011). Ethnomedicinal studies on the plant resources of east Anatolia, Turkey. Procedia-Soc. Behav. Sci..

[12] Ghafari S., Tavakoli Z., Shirooyeh P., Meybodi R.N., Behmanesh E., Mokaberinejad R., Tansaz M., Fahimi S. (2018). The herbal medicine proposed by Iranian Traditional Medicine (Persian Medicine) for treatment of primary Dysmenorrhea: A Review. Tradit. Integr. Med..

[13] Hosokawa K., Fukunaga Y., Fukushi E., Kawabata J. (1995). Acylated anthocyanins from red Hyacinthus orientalis. Phytochemistry.

[14] Karaman S., Kocabas Y.Z. (2001). Traditional medicinal plants of K. Maras (Turkey). Science.

[15] Hosokawa K., Fukunaga Y., Fukushi E., Kawabata J. (1996). Acylated anthocyanins in red flowers of Hyacinthus orientalis regenerated in vitro. Phytochemistry.

[16] Lin B.W., Gong C.C., Song H.F., Cui Y.Y. (2017). Effects of anthocyanins on the prevention and treatment of cancer. Br. J. Pharmacol..

[17] Yin Z., Zhang W., Feng F., Zhang Y., Kang W. (2014). α-Glucosidase inhibitors isolated from medicinal plants. Food Sci. Hum. Wellness.

[18] Kayıran S.D., Özkan E.E. (2017). The Ethnobotanical Uses of Hyacinthaceae Species Growing in Turkey and a Review of Pharmacological Activities. http://nopr.niscair.res.in/handle/123456789/40121.

[19] Talib W.H., Al Kury L.T. (2018). Parthenolide inhibits tumor-promoting effects of nicotine in lung cancer by inducing P53-dependent apoptosis and inhibiting VEGF expression. Biomed. Pharmacother..

[20] Musumeci G., Loreto C., Carnazza M.L., Martinez G. (2011). Characterization of apoptosis in articular cartilage derived from the knee joints of patients with osteoarthritis. Knee Surg. Sports Traumatol. Arthrosc..

[21] Jin Z., El-Deiry W.S. (2005). Overview of cell death signaling pathways. Cancer Biol. Ther..

[22] Talib W.H. (2017). Regressions of breast carcinoma syngraft following treatment with piperine in combination with thymoquinone. Sci. Pharm..

[23] Misra P., Khaliq T., Dixit A., SenGupta S., Samant M., Kumari S., Kumar A., Kushawaha P.K., Majumder H.K., Saxena A.K. (2008). Antileishmanial activity mediated by apoptosis and structure-based target study of peganine hydrochloride dihydrate: An approach for rational drug design. J. Antimicrob. Chemother..

[24] Jia S.-S., Xi G.-P., Zhang M., Chen Y.-B., Lei B., Dong X.-S., Yang Y.-M. (2013). Induction of apoptosis by D-limonene is mediated by inactivation of Akt in LS174T human colon cancer cells. Oncol. Rep..

[25] Messeha S.S., Zarmouh N.O., Asiri A., Soliman K.F. (2020). Rosmarinic acid-induced apoptosis and cell cycle arrest in triple-negative breast cancer cells. Eur. J. Pharmacol..

[26] Fong Y., Tang C.-C., Hu H.-T., Fang H.-Y., Chen B.-H., Wu C.-Y., Yuan S.-S., Wang H.-M.D., Chen Y.-C., Teng Y.-N. (2016). Inhibitory effect of trans-ferulic acid on proliferation and migration of human lung cancer cells accompanied with increased endogenous reactive oxygen species and β-catenin instability. Chin. Med..

[27] Basli A., Belkacem N., Amrani I. (2017). Health Benefits of Phenolic Compounds against Cancers. Phenolic Compounds—Biological Activity.

[28] Al Obaydi M.F., Hamed W.M., Al Kury L.T., Talib W.H. (2020). *Terfezia boudieri*: A desert truffle with anticancer and immunomodulatory activities. Front. Nutr..

[29] Talib W.H. (2017). Consumption of garlic and lemon aqueous extracts combination reduces tumor burden by angiogenesis inhibition, apoptosis induction, and immune system modulation. Nutrition.

[30] Giannoulia-Karantana A., Vlachou A., Polychronopoulou S., Papassotiriou I., Chrousos G.P. (2006). Melatonin and immunomodulation: Connections and potential clinical applications. Neuroimmunomodulation.

[31] Yamamoto M., Kamigaki T., Yamashita K., Hori Y., Hasegawa H., Kuroda D., Moriyama H., Nagata M., Ku Y., Kuroda Y. (2009). Enhancement of anti-tumor immunity by high levels of Th1 and Th17 with a combination of dendritic cell fusion hybrids and regulatory T cell depletion in pancreatic cancer. Oncol. Rep..

[32] Eggermont L.J., Paulis L.E., Tel J., Figdor C.G. (2014). Towards efficient cancer immunotherapy: Advances in developing artificial antigen-presenting cells. Trends Biotechnol..

[33] Del Toro-Arreola S., Flores-Torales E., Torres-Lozano C., Del Toro-Arreola A., Tostado-Pelayo K., Ramirez-Dueñas M.G., Daneri-Navarro A. (2005). Effect of D-limonene on immune response in BALB/c mice with lymphoma. Int. Immunopharmacol..

[34] Lappas C.M., Lappas N.T. (2012). D-Limonene modulates T lymphocyte activity and viability. Cell. Immunol..

[35] Bhardwaj J., Chaudhary N., Seo H.-J., Kim M.-Y., Shin T.-S., Kim J.-D. (2014). Immunomodulatory effect of tea saponin in immune T-cells and T-lymphoma cells via regulation of Th1, Th2 immune response and MAPK/ERK2 signaling pathway. Immunopharmacol. Immunotoxicol..

[36] Becker K., Schwaiger S., Waltenberger B., Fuchs D., Pezzei C., Schennach H., Stuppner H., Gostner J. (2018). Immunomodulatory Effects of Diterpene Quinone Derivatives from the Roots of Horminum pyrenaicum in human PBMC. Oxidative Med. Cell. Longev..

[37] Heo S.-K., Noh E.-K., Yoon D.-J., Jo J.-C., Koh S., Baek J.H., Park J.-H., Min Y.J., Kim H. (2015). Rosmarinic acid potentiates ATRA-induced macrophage differentiation in acute promyelocytic leukemia NB4 cells. Eur. J. Pharmacol..

[38] Ahn J., Kil D., Kong C., Kim B. (2014). Comparison of oven-drying methods for determination of moisture content in feed ingredients. Asian Australas. J. Anim. Sci..

[39] Talib W.H., Mahasneh A.M. (2010). Antiproliferative activity of plant extracts used against cancer in traditional medicine. Sci. Pharm..

[40] Sabbah D.A., Al-Tarawneh F., Talib W.H., Sweidan K., Bardaweel S.K., Al-Shalabi E., Zhong H.A., Abu Sheikha G., Abu Khalaf R., Mubarak M.S. (2018). Benzoin schiff bases: Design, synthesis, and biological evaluation as potential antitumor agents. Med. Chem..

[41] Chen J.-R., Yang Z.-Q., Hu T.-J., Yan Z.-T., Niu T.-X., Wang L., Cui D.-A., Wang M. (2010). Immunomodulatory activity in vitro and in vivo of polysaccharide from Potentilla anserina. Fitoterapia.

[42] Boothapandi M., Ramanibai R. (2016). Immunomodulatory activity of Indigofera tinctoria leaf extract on in vitro macrophage responses and lymphocyte proliferation. Int. J. Pharm. Pharm. Sci..

[43] Moretão M.P., Buchi D.F., Gorin P.A., Iacomini M., Oliveira M.B.M. (2003). Effect of an acidic heteropolysaccharide (ARAGAL) from the gum of *Anadenanthera colubrina* (*Angico branco*) on peritoneal macrophage functions. Immunol. Lett..

[44] Liu Z., Xing J., Huang Y., Bo R., Zheng S., Luo L., Niu Y., Zhang Y., Hu Y., Liu J. (2016). Activation effect of Ganoderma lucidum polysaccharides liposomes on murine peritoneal macrophages. Int. J. Biol. Macromol..

[45] Rainard P. (1986). A colorimetric microassay for opsonins by reduction of NBT in phagocytosing bovine polymorphs. J. Immunol. Methods.

[46] Falah R.R., Talib W.H., Shbailat S.J. (2017). Combination of metformin and curcumin targets breast cancer in mice by angiogenesis inhibition, immune system modulation and induction of p53 independent apoptosis. Adv. Med. Oncol..

[47] Akyüz M. (2013). Nutritive value, flavonoid content and radical scavenging activity of the truffle (*Terfezia boudieri* Chatin). J. Soil Sci. Plant Nutr..

[48] Akhila J.S., Shyamjith D., Alwar M. (2007). Acute toxicity studies and determination of median lethal dose. Curr. Sci..

[49] Agrawal N., Bettegowda C., Cheong I., Geschwind J.-F., Drake C.G., Hipkiss E.L., Tatsumi M., Dang L.H., Diaz L.A., Pomper M. (2004). Bacteriolytic therapy can generate a potent immune response against experimental tumors. Proc. Natl. Acad. Sci. USA.

