# Supplementary material for: Immunomodulatory and Anticancer Activities of Hyacinthus orientalis L.: An In Vitro and In Vivo Study
Source: Plants (Basel). 2021 Mar 24;10(4):617. doi: 10.3390/plants10040617 (PMC8063964; doi:10.3390/plants10040617)
Supplement: Supplementary file 1 [file plants-10-00617-s001.pdf]

S1

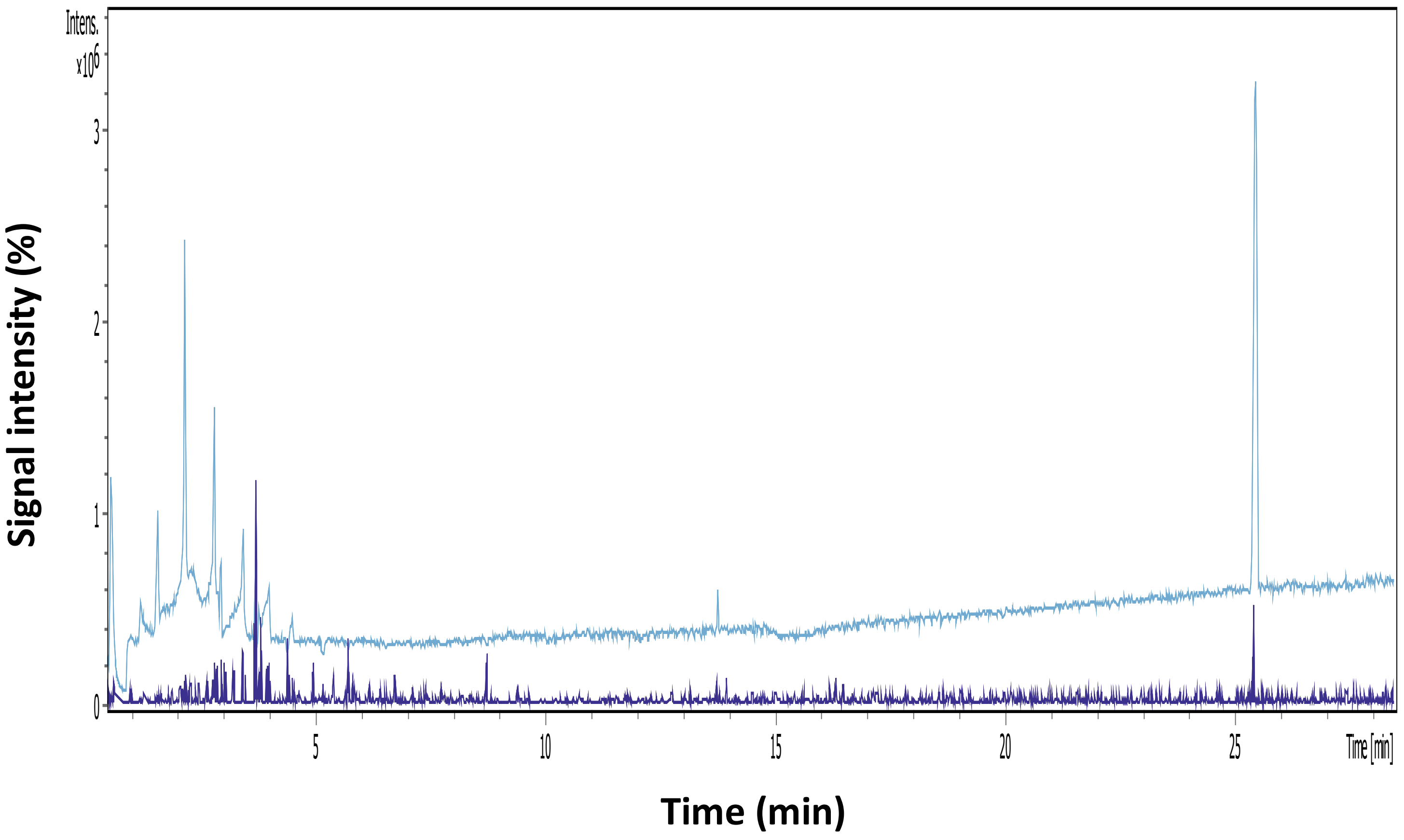

S1: LC-MS Chromatogram obtained from *H. orientalis* aqueous fraction

S2

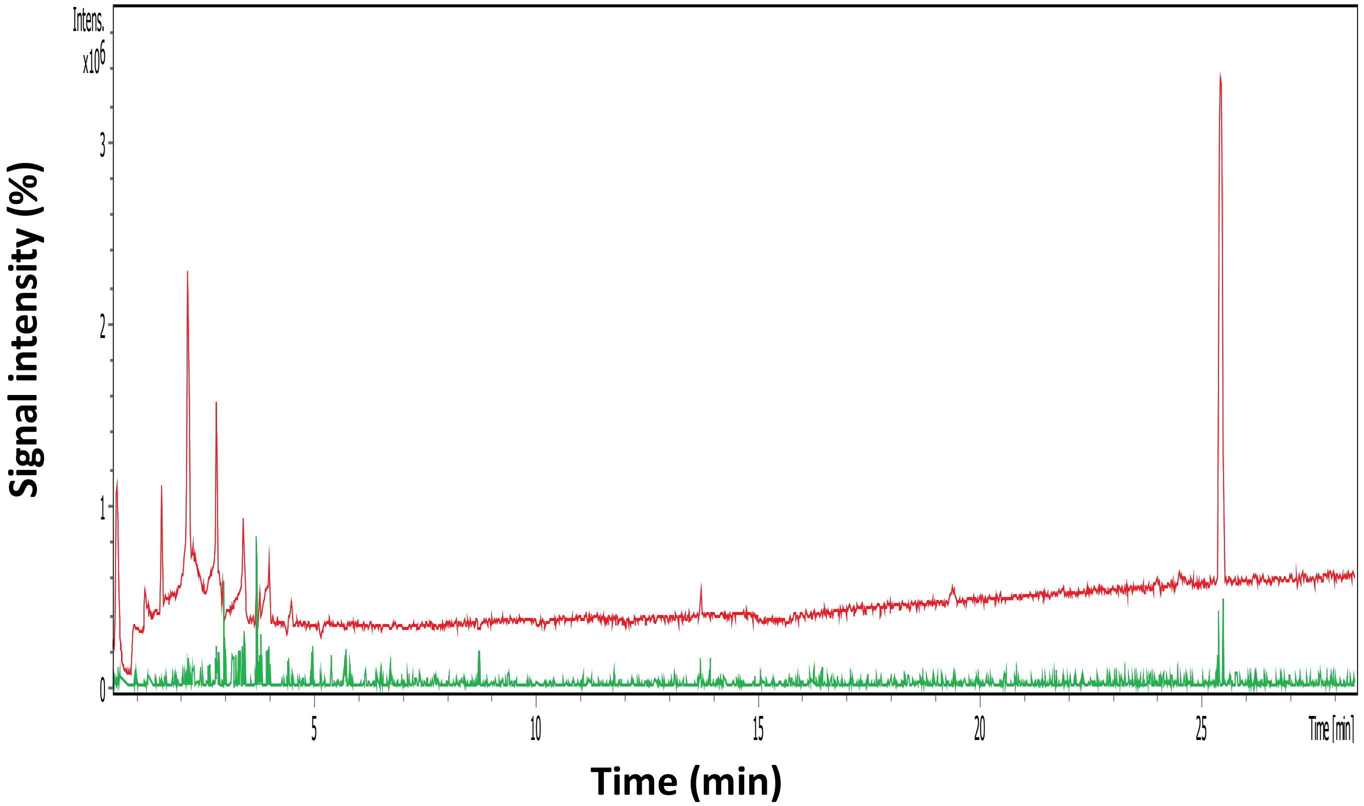

S2: LC-MS Chromatogram obtained from *H. orientalis* hydroalcoholic extract

S3

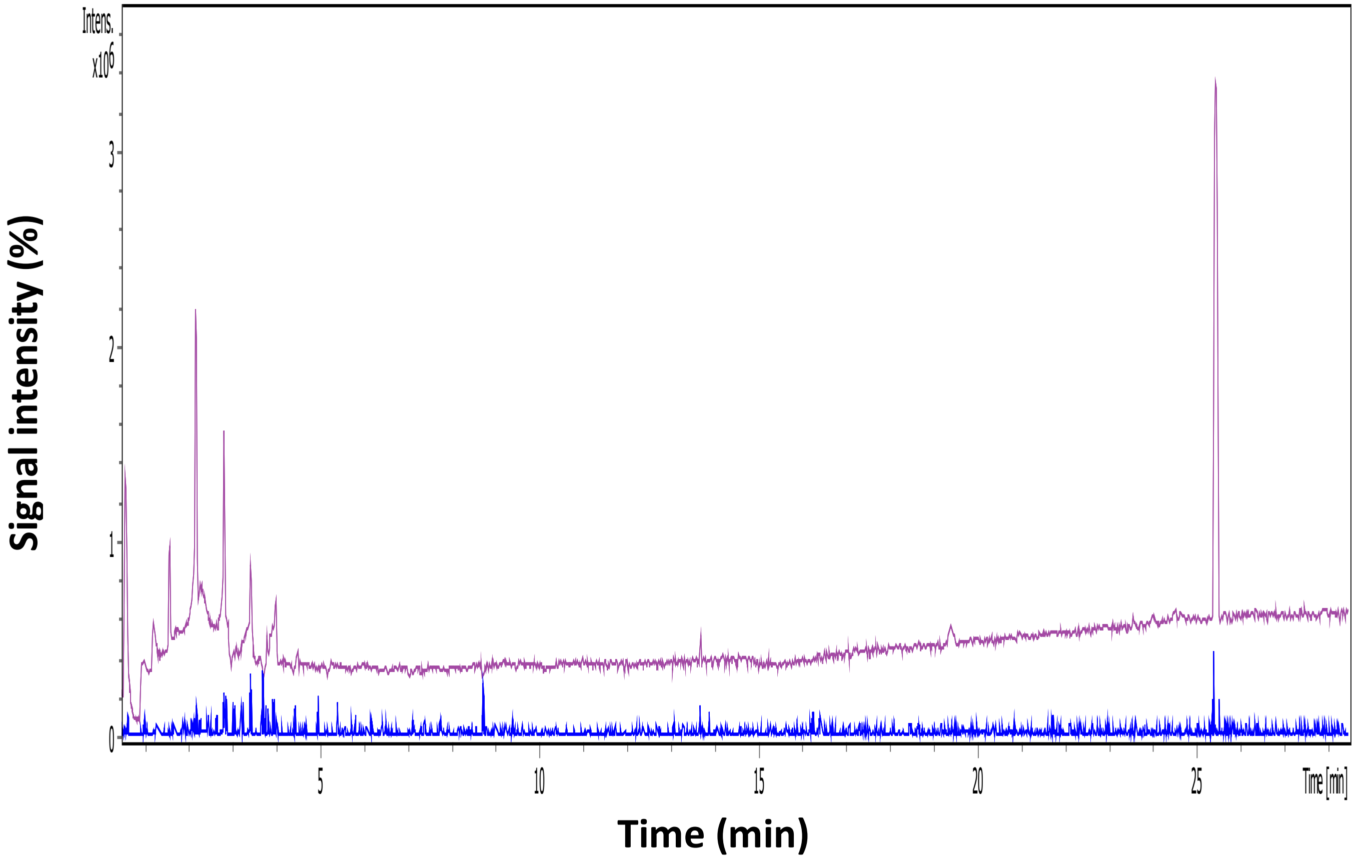

S3: LC-MS Chromatogram obtained from *H. orientalis* chloroform fraction

S4

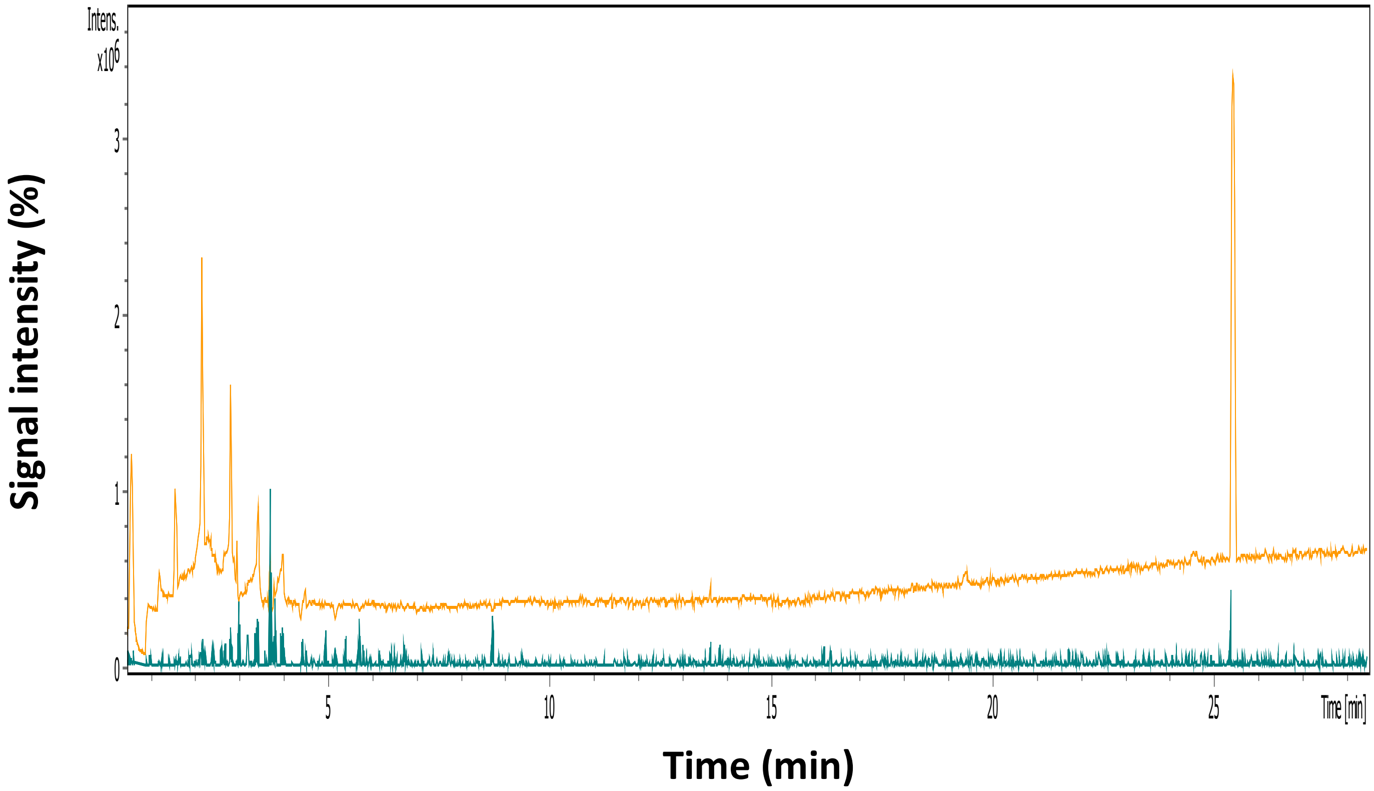

S4: LC-MS Chromatogram obtained from *H. orientalis* aqueous methanol fraction

S5

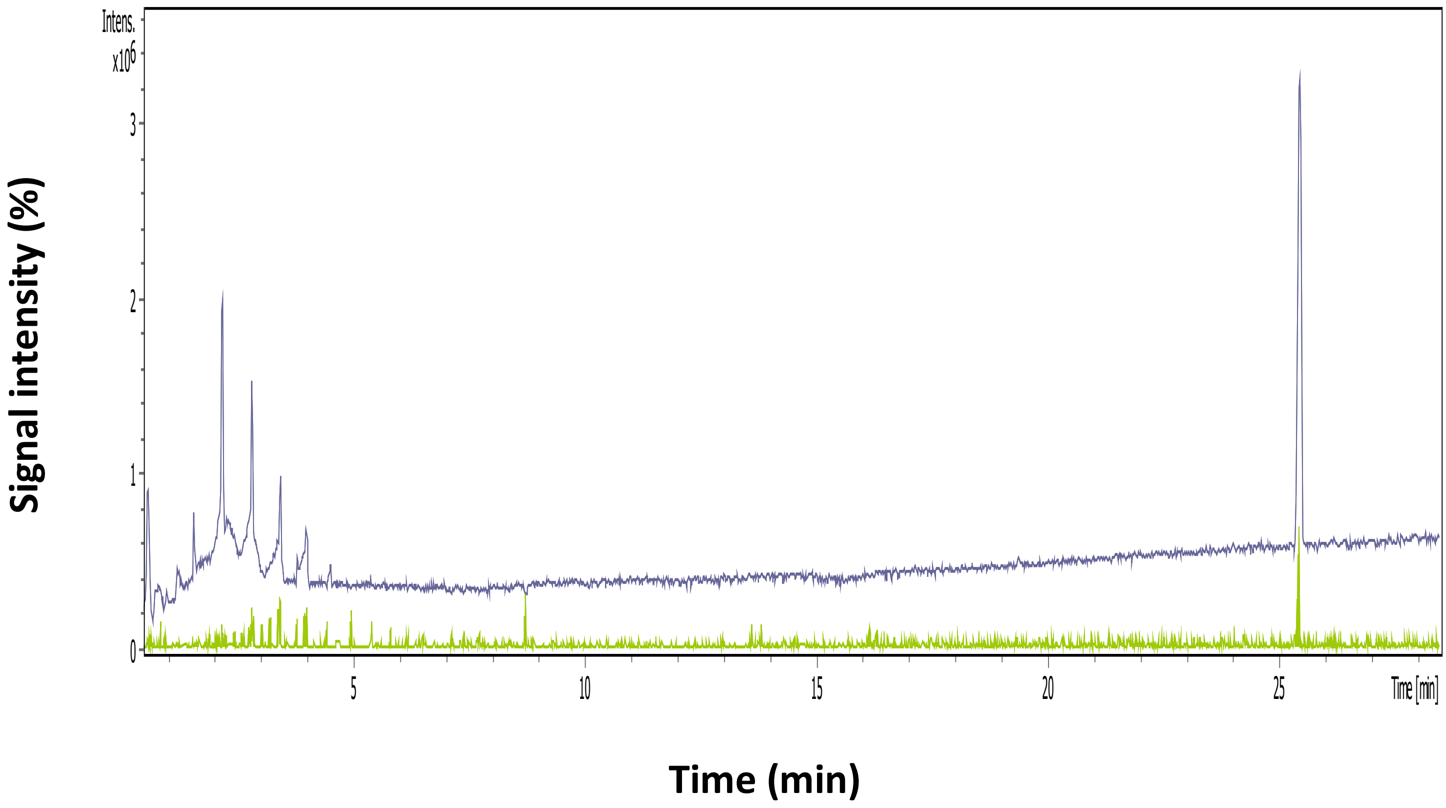

S5: LC-MS Chromatogram obtained from *H. orientalis* *n*-hexane fraction
